# Textural feature based intelligent approach for neurological abnormality detection from brain signal data

**DOI:** 10.1371/journal.pone.0277555

**Published:** 2022-11-14

**Authors:** Md. Nurul Ahad Tawhid, Siuly Siuly, Kate Wang, Hua Wang

**Affiliations:** 1 Institute for Sustainable Industries & Liveable Cities, Victoria University, Melbourne, Victoria, Australia; 2 School of Health and Biomedical Sciences, RMIT University, Melbourne, Victoria, Australia; Sejong University, KOREA, REPUBLIC OF

## Abstract

The diagnosis of neurological diseases is one of the biggest challenges in modern medicine, which is a major issue at the moment. Electroencephalography (EEG) recordings is usually used to identify various neurological diseases. EEG produces a large volume of multi-channel time-series data that neurologists visually analyze to identify and understand abnormalities within the brain and how they propagate. This is a time-consuming, error-prone, subjective, and exhausting process. Moreover, recent advances in EEG classification have mostly focused on classifying patients of a specific disease from healthy subjects using EEG data, which is not cost effective as it requires multiple systems for checking a subject’s EEG data for different neurological disorders. This forces researchers to advance their work and create a single, unified classification framework for identifying various neurological diseases from EEG signal data. Hence, this study aims to meet this requirement by developing a machine learning (ML) based data mining technique for categorizing multiple abnormalities from EEG data. Textural feature extractors and ML-based classifiers are used on time-frequency spectrogram images to develop the classification system. Initially, noises and artifacts are removed from the signal using filtering techniques and then normalized to reduce computational complexity. Afterwards, normalized signals are segmented into small time segments and spectrogram images are generated from those segments using short-time Fourier transform. Then two histogram based textural feature extractors are used to calculate features separately and principal component analysis is used to select significant features from the extracted features. Finally, four different ML based classifiers are used to categorize those selected features into different disease classes. The developed method is tested on four real-time EEG datasets. The obtained result has shown potential in classifying various abnormality types, indicating that it can be utilized to identify various neurological abnormalities from brain signal data.

## Introduction

Recent years have seen extensive research on brain signal data, notably employing electroencephalogram (EEG) data because of its crucial role in applications for health and medicine. [1–5]. Efficient and effective analysis of EEG signal is useful for various purposes like neurological diseases diagnosis and treatment [6–11], brain computer interface [12–15], emotion/fatigue detection [16–18], sleep stage detection [19] etc. EEG captures the electrical activity of the brain as a time-series data with dynamic, non-stationary and aperiodic in nature. It is a large volume of data that contains patterns related to the subject’s mental health state [7, 20]. Currently, accurate and efficient analysis of these large-scale aperiodic and non-stationary EEG signals is a challenging task [10]. Data mining system allows the extraction of important biomarkers from the brain signal data and use those biomarkers for automatically classifying brain states into different types of abnormalities by creating computer aided diagnosis (CAD) system.

Artificial intelligence can help healthcare providers with a wide range of patient treatment and intelligent healthcare systems [4]. In recent years, several frameworks have been developed for analyzing and classifying large scale EEG signal data [10, 20–33]. Most of these studies have considered identifying one neurological abnormality (two class classification) from EEG data. Developing CAD system using those methods for multiple neurological disorders will require multiple separate systems for each diseases. It will be costly and also time consuming for checking multiple disorders. Few researchers have attempted classifying two neurological abnormalities from healthy control (HC) participants (three classes) in the same method, as the authors of [34–36] worked in detecting mild cognitive impairment (MCI) and Alzheimer’s disease (AD) patients from HC subjects. Similarly, authors of [37, 38] have developed a system to classify autism spectrum disorder (ASD) and epilepsy (EP) from HC subjects using EEG signal data. To the best of our knowledge, no research has looked into the detection of more than two anomalies in a single data mining framework from HC subjects. This is due to the vast volume of EEG signal data and overlap between the biomarkers for various disorders in the signal data.

As a result, specific data mining approaches are needed to execute classification on this type of overlapping feature-based data into multiple classes. Additionally, a single mining framework is required to execute the classification operation and find various types of abnormalities from the EEG signal with related abnormality attributes. The motivation of this study is to fill this knowledge gap by developing a brain signal data mining methodology for categorizing into several anomaly classes based on the biomarkers displayed in the visual representations of the signal data.

Broadly, the data mining process of the existing studies can be divided into two steps: feature extraction from the signal data and classification of the extracted features using different classifiers. Majority of the researches used various statistical information as signal features and then classified those features using different classifiers. When the data volume is high, these conventional methods cannot be used frequently to extract substantial and differentiating features from EEG data [39]. Additionally, when statistical features for larger recording (long-term) data are extracted, it is possible to overlook the short-term changes in signal characteristics that are crucial for anomalies identification [39]. Visual representation of small signal segments can solve this issue as it uses the raw signal data for producing visual representation and works on small segments of the data [10, 39].

To accomplish the above mentioned aim, in our recent work [40], we have introduced a time-frequency (T-F) spectrogram image based data mining technique for brain signal data specially EEG to identify four different neurological abnormalities named: ASD, EP, Parkinson’s disease (PD), and schizophrenia (SZ) from HC subjects (five class). Spectrogram images are used for 2D visualization of EEG signals in time-frequency (T-F) domain and describes the nonstationary characteristics of the signal data [10]. The frequency spectrum of the spectrogram image changes over time, and the colors on the image reflect various energy values [39]. When compared to other feature extraction techniques, spectrogram images contain more unidentified EEG signal characteristics and may perform better in a classification algorithm [39]. Spectrogram images have previously been utilized for identifying patients from healthy controls (HC) for various neurological disorders such as epilepsy [41], epileptic seizures [21], ASD [39] and schizophrenia [42], and achieved good classification performance, which drives us to apply them in this study.

In this work, we have extended our recent work [40] using a different textural feature extractor. At first, the brain signal data are filtered for removing noise and artifacts from the signal data. Then the signals are segmented into small time frame window and spectrogram plotting images are generated from those small chunks using short-time fourier transform (STFT). In [40], histogram based textural features are extracted from those images using completed CENsus TRanform hISTogram (cCENTRIST), a histogram-based feature extraction technique proposed by Dey *et al*. [43] and performed well on garments texture classification. In this work, we have used another histogram-based feature extractor developed by Dey *et al*. [43] named ternary CENsus TRanform hISTogram (tCENTRIST) that performed well on spectrogram image classification [39]. After that, principal component analysis (PCA) is used to reduce the dimension of the extracted features. Finally, four ML based classifiers namely: support vector machine (SVM), *k*-nearest neighbor (*k*NN), random forest (RF) and Linear Discriminant Analysis (LDA) are used to categorize the reduced extracted features.

Following are the significant contributions of this study:

1. A single unified ML based framework is designed to classify multiple neurological abnormality from brain signal data
2. Two distinct feature extractors in combination with four different ML based classifiers are examined.
3. Validate the proposed framework using four EEG signal datasets from four different neurological abnormalities.
4. Obtain improved performance for the multi-disease classification process compared to the existing methods.

The remainder of the paper is laid out as follows: The proposed method’s workflow is described in depth in Section. Section 7 provides a detailed description of the datasets used in this study and evaluation parameters. The experimental results with visual and tabular representations are given in Section 7. Finally, Section 7 closes the paper with concluding remarks.

## Workflow of the proposed framework

**Fig 1:**
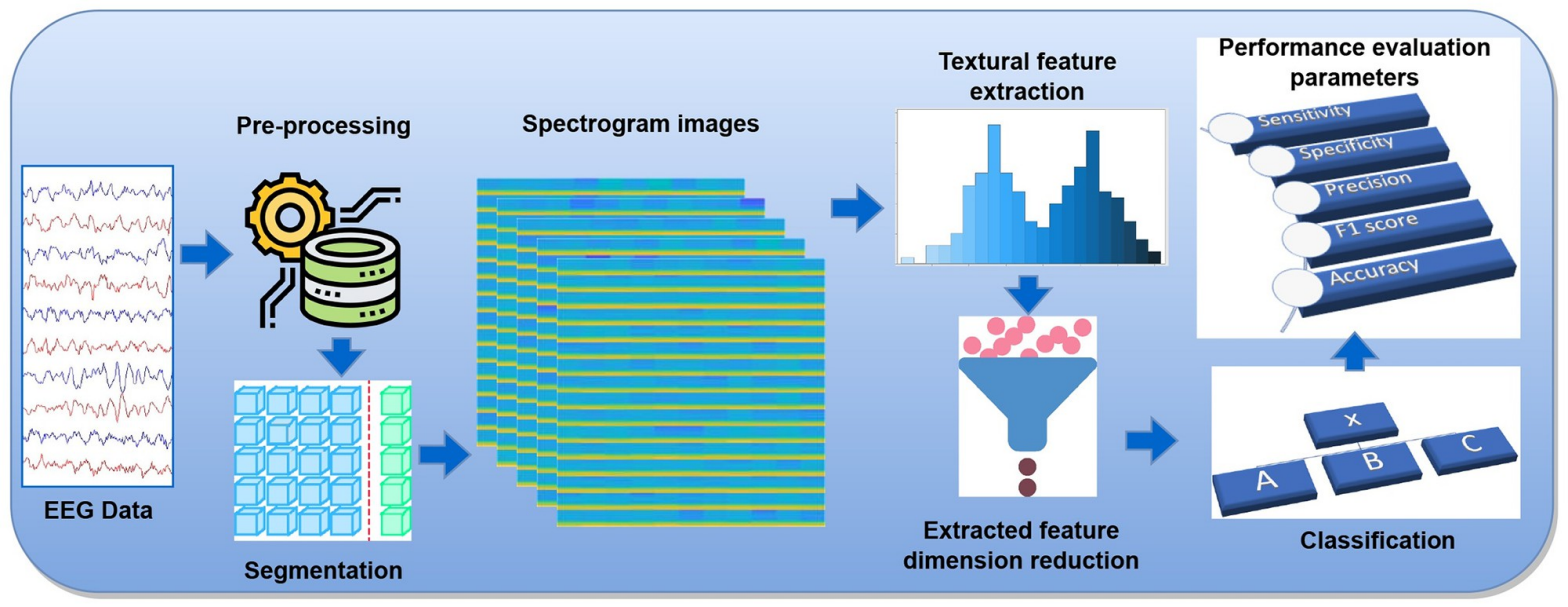
Overview of the proposed framework.

In this study, we have used T-F based spectrogram image for classification of brain signal data using cCENTRIST and tCENTRIST based feature extraction techniques with four different machine learning based classification approaches namely: *k*NN, SVM, RF and LDA. The proposed process consists of several steps: firstly, the raw brain signal data are pre-processed for artifact removal. Then the signals are segmented into small time frame and generated spectrogram images from those segments using STFT. After that, features are extracted from those images using cCENTRIST and tCENTRIST based technique and the dimensions of the extracted features are reduced using PCA. Finally, four different classifiers namely: *k*NN, SVM, RF and LDA are used for classifying the spectrogram images into different classes. An overview of the proposed method is given in Fig 1. Details of these steps are discussed in below subsections.

### Pre-processing the brain signal data

In this step, we have pre-processed the brain signal data for removing the noise and artifacts introduced by the recording environment and the muscle movement of the subject during the recording time. These filtering processes are done due to some noise and artifacts are very much similar to some disease related signal patterns and may mislead the diagnosis process [44]. To perform the filtering, at first, we used the common average referencing (CAR) technique to remove the common noise and signals from all channels by removing the average signal from all electrodes. After that, artifacts introduced by muscle activity, eye movement and external noise are removed by passing the signal into a low pass infinite impulse response (IIR) filter with a cutoff frequency of 40Hz. Finally, the signals are normalized to a distribution of zero mean and a variance of one to reduce the individual signal differences and computational complexity.

### Spectrogram image generation

In this step, the pre-processed signal data are converted into spectrogram images. We have done this in two steps: at first the brain signal data are segmented into small chunks of three seconds (3s) to increase the dataset size and as well as extract maximum number of features from the small signal segments [45]. In this segmentation process original signals are segmented into small data chunks and given the level of original data, which makes an increase in the sample size.

After that, spectrogram images are generated from those small chunks using STFT based spectrogram plotting technique. Spectrogram is a popularly used technique for time-frequency domain analysis of EEG signal data. STFT converts the the time varying EEG signal to a two-dimensional matrix with time and frequency axes. In order to calculate the STFT, at first, the signal is divided into a number of short-time overlapping windowed blocks [46]. Then, in order to ensure continuity between the first and last points in the frames and avoid the leakage effect on the spectrum, a hamming window approach is used. Then, each segment’s fourier transform (FT) is computed in order to obtain its own local frequency spectrum. The STFT of a signal *x*(*t*) is calculated using the below Eq 1:

$$\begin{array}{c} STFT\left\{x\left(t\right)\right\}=X\left(\tau ,\omega \right)=\int_{-\infty }^{\infty }x\left(t\right)w\left(t-\tau \right)e^{-i\omega t}dt \end{array}$$ (1)

Here, *ω* is the signal frequency, *w*(*τ*) is the nonzero window function and *X*(*τ*, *ω*) is the FT of the product *x*(*t*)*w*(*t* − *τ*), reflecting the signal’s phase and amplitude with time and frequency. STFT is frequently visualized by its spectrogram, which is an intensity representation of STFT magnitude over time. These images are further used for feature extraction and classification process for this study.

### Feature extraction and dimension reduction

In this step, features from the spectrogram images are extracted and dimension of the extracted features are reduced for classification process. We have used two texture based feature extractor named completed CENsus TRanform hISTogram (cCENTRIST) and ternary CENsus TRanform hISTogram (tCENTRIST) proposed by Dey *et al*. [43]. cCENTRIST was developed by replacing Linear Binary Pattern (LBP) of CENsus TRanform hISTogram (CENTRIST) [47] with Completed Local Binary Pattern (CLBP) while tCENTRIST was developed by replacing LBP with Local Ternary Pattern (LTP) and both of those feature extractor performed well on classification of garments texture [43] and face image based gender identification [48]. A brief description CENTRIST, cCENTRIST and tCENTRIST are given in below sections:

#### CENsus TRanform hISTogram (CENTRIST)

**Fig 2:**
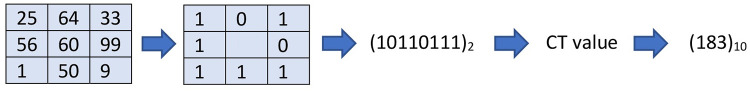
Census Transform (CT) calculation process used in CENTRIST. Here, a bit 1 is set in the relevant spot if the central pixel is larger than (or equal to) one of its neighbors. If not, bit 0 is set.

CENTRIST is a non-parametric local transform approach built on the idea of Census Transform (CT) [49], which maps a pixel by comparing intensity values with its eight neighboring pixels and generates an eight bit string (CT values). This approach is similar to LBP except that LBP performs interpolation for corner pixels but CENTRIST considers those pixels as is. A sample CT calculation process is given in Fig 2.

In order to collect both the local and global information for an image, CENTRIST creates a histogram from the CT values of image patches. They also employed spatial representation based on the Spatial Pyramid Matching (SPM) technique, which divides a picture into smaller parts and incorporates correspondence results in those regions to enhance recognition. Finally, PCA is used to reduce the dimensions of the extracted features of CENTRIST.

#### Completed CENTRIST (cCENTRIST)

In this texture extractor, the authors have used CLBP for generating CT values in place of LBP in CENTRIST. When comparing a pixel to its neighbors, CLBP considers both the magnitude (CLBP_M) and the signs (CLB_S) of the differences. Additionally, it uses global thresholding to provide a binary code (CLBP_C) for the center pixel. An uniform and rotation-invariant CT code is generated by CLBP using sign, magnitude, and center-pixel information.

For an image of size 3x3, differences (*d*_*p*_) have two different components calculated from the differences between each neighboring pixel to the central pixel using Eq 2, where, *s*_*p*_ and *m*_*p*_ are the sign and magnitude part of the differences *d*_*p*_.

$$\begin{array}{c} d_{p}=S_{P}\times m_{p}\;and\{\begin{array}{cc} S_{P}= & sign\left(d_{p}\right),\;\left[1\;if\;d_{P}\geq 0,\;else\;-1\right]\\ m_{p}= & \left|d_{p}\right| \end{array} \end{array}$$ (2)

If *P* and *R* are the neighbor number and radius of LBP code, respectively, then *CLBP*_*S*_*P*,*R*_, *CLBP*_*M*_*P*,*R*_ and *CLBP*_*C*_*P*,*R*_ are calculated using the Eqs 3–5 as follows:

$$\begin{array}{c} CLBP\_C_{P,R}=t\left(g_{c},c\right),t\left(x,c\right)=\{\begin{array}{cc} 1, & x\geq c\\ 0, & x<c \end{array} \end{array}$$ (3)

$$\begin{array}{c} CLBP\_S_{P,R}=\sum_{p=0}^{P-1}s\left(g_{p}-g_{c}\right)2^{p},s\left(x\right)=\{\begin{array}{cc} 1, & x\geq 0\\ 0, & x<0 \end{array} \end{array}$$ (4)

$$\begin{array}{c} CLBP\_M_{P,R}=\sum_{p=0}^{P-1}t\left(m_{p},c\right)2^{p},t\left(x,c\right)=\{\begin{array}{cc} 1, & x\geq c\\ 0, & x<c \end{array} \end{array}$$ (5)

Here *c* is a threshold calculated as the average of the whole image, *g*_*c*_ is the gray value of the center pixel and *g*_*p*_(*p* = 0, 1, …., *P* − 1) is the neighboring pixel’s gray value on a circle with radius R. Finally, a 3D histogram is generated as CT value using *CLBP*_*S*_*P*,*R*_, *CLBP*_*M*_*P*,*R*_ and *CLBP*_*C*_*P*,*R*_ and PCA is applied to reduce the dimension of the feature vector. Algorithm 1 describes the process of cCENTRIST.

**Algorithm 1**: Feature extraction and dimension reduction using cCENTRIST and PCA

**Input**: Spectrogram image *I*

**Output**: Dimension reduced feature vector of *I*

1 Initialization;

2 Calculate level 2 Spatial Pyramid (SP) for the image *I*

3 **for** *each block of SP* **do**

4  (a) Calculate *CLBP*_*C*_*P*,*R*_, *CLBP*_*S*_*P*,*R*_ and *CLBP*_*M*_*P*,*R*_ using Eqs 3, 4 and 5, respectively

5  (b) Concatenate all histograms from each to form a single histogram feature block

6 Apply PCA to extract M feature points from the extracted features

#### Ternary CENTRIST (tCENTRIST)

It used LTP in place of LBP in CENTRIST that introduces a new bit to handle the fluctuations intensity. For an image of size 3x3, LTP produces a ternary code for each central pixel c using the Eq 6:

$$\begin{array}{c} LTP_{P,R}=\sum_{p=0}^{P-1}q\left(g_{p}-g_{c}\right)3^{p},q\left(a\right)=\{\begin{array}{cc} 1 & ifa\geq \mu \\ -1 & ifa<\mu \\ 0 & otherwise \end{array} \end{array}$$ (6)

where, *μ* is a threshold value of ±5 and *g*_*p*_, *g*_*c*_, *P*, *R* are defined in Eqs 3–5. After calculating the LTP values, two histograms are generated using the upper and lower code of LTP and finally concatenated to build a single histogram. Afterwards, PCA is applied to reduce the dimension of the feature vector. Algorithm 2 describes the process of tCENTRIST.

**Algorithm 2**: Feature extraction and dimension reduction using tCENTRIST and PCA

**Input**: Spectrogram image *I*

**Output**: Dimension reduced feature vector of *I*

1 Initialization;

2 Calculate level 2 Spatial Pyramid (SP) for the image *I*

3 **for** *each block of SP* **do**

4  (a) Calculate *LTP* value using Eq 6.

5  (b) Construct a histogram using the LTP value;

6 Concatenate all histograms from each to form a single histogram feature block

7 Apply PCA to extract M feature points from the extracted features

Both of those feature extractor uses a Spatial Pyramid (SP) structure that breaks the images into pyramid structure blocks. Later, to reduce the computational complexity and to use significant features, PCA is used to reduce the dimension of the extracted features. Finally, four ML based classifiers are used to classify those reduced features into different classes.

### Classification of the extracted features

The extracted features of the previous step are classified in this step using different ML based techniques. In this study, we have used two different histogram based textural feature extractor named cCENTRIST and tCENTRIST along with four different ML based classifiers namely: RF, *k*NN, LDA and SVM for classifying the spectrogram images. Finally, these classifiers perform a multi-class classification for different neurological disorders and their performance are evaluated using different evaluation techniques.

- **Support Vector Machine (SVM)**: Currently SVM is an efficient and effective classifier in the field of detecting abnormalities from brain signal data. It excels at dealing with high-dimensional and non-linear data. In this study, we used the same LibSVM [50] as the authors of cCENTRIST and tCENTRIST [43] used, which is SVM with following linear kernel function, *K(x, y)*:
  $$\begin{array}{c} K\left(x,y\right)=x^{T}y \end{array}$$ (7)
  Here, kernel function constructed from the dot product of two invariant x and y.
- ***k*-Nearest Neighbor (*k*NN)**: The Second classifier we have tested is *k*NN, which is simple and robust for large scale datasets. It carries out the classification operation based on the frequent class of its closest neighbors in the feature space [51]. In *k*NN based classification, we have tested for 10 different *k* values (1 to 10) with Euclidean distance metrics as defined follows:
  $$\begin{array}{c} D\left(x_{y},s\right)=\sqrt{\sum_{i=1}^{n}{\left(s_{i}-x_{y}\right)}^{2}} \end{array}$$ (8)
  Here, s denotes the training set and y is the unknown test data.
- **Random Forest (RF)**: Next classifier we have tested is RF introduced by Leo Breiman [52], which is an ensemble learning method with a collection of multiple decision trees. We have used entropy as impurity metrics for building RF which is defined as follows:
  $$\begin{array}{c} Entropy,I_{E}=-\sum_{i=1}^{n}p_{i}{\text{log}}_{2}p_{i} \end{array}$$ (9)
  Here, *p*_*i*_ refers to the probability of class *c*_*i*_ in the data sample.
- **Linear Discriminant Analysis (LDA)**: Fourth and final classifier we have used is LDA that performed well in many classification tasks like emotional speech recognition, multimedia information retrieval, face recognition, image identification, etc. [53]. For each class *c* with a mean *μ*_*c*_ and covariance *Σ*, LDA is calculated as follows:
  $$\begin{array}{c} y_{c}=x^{T}\Sigma^{-1}\mu_{c}-\frac{1}{2}\mu_{c}^{T}\Sigma^{-1}\mu_{c}+log\frac{n_{c}}{n} \end{array}$$ (10)
  where *x* is the test instance, *n*_*c*_ and *n* are the number of instances in class *c* and in whole dataset, respectively. *x* is classified with the highest *y*_*c*_ values.

## Performance evaluation materials and parameters

To validate the proposed model, we have used EEG brain signal data from four different neurological disorders namely: ASD, EP, PD and SZ. We have used these four datasets to perform a five class (ASD vs EP vs PD vs SZ vs HC) classification using the proposed method. Performance of the proposed method is evaluated using different evaluation matrices that are popular in this field of study. Details of the datasets and evaluation matrices are discussed in below:

### Datasets

In this study, we have used four publicly available datasets of four different neurological abnormalities (ASD, EP, PD, SZ) for validating the proposed brain signal data mining system. A brief description of those datasets are given below:

- For ASD, we have used the King Abdulaziz University (KAU) Hospital in Jeddah, Saudi Arabia [54]. The dataset contains sixteen subjects (twelve ASD and four HC subjects) with no record of cognitive disorders. For EEG recording, they used 16 channels (FP1, FP2, F3, F4, F7, F8, T3, T5, C4, Fz, Cz, Pz, C3, O1, Oz and O2) from standard 10-20 international system. Resting state EEG data was recorded from each of the subjects and sampled at a frequency of 256Hz.
- Epilepsy dataset was collected in Universidade Federal do Para, Brazil [55]. This dataset contains 14 subjects’ (7 patients and 7 HC) EEG signals. Resting state EEG data was recorded from 20 channels (Fp1, Fp2, F3, F4, F7, F8, C3, C4, T3, T4, P3, P4, T5, T6, O1, O2, FZ, CZ, PZ, OZ) at a sampling rate of 256Hz.
- The third dataset we have used is for parkinson’s disease collected from University of Iowa, Iowa City, Iowa, United States [25]. It has 28 subjects from two groups (14 PD patients and 14 control subjects). Resting state EEG data with a sampling rate of 500Hz was collected from 64 channels for PD patients.
- Finally, for schizophrenia, we used the dataset from Institute of Psychiatry and Neurology in Warsaw, Poland [24]. This dataset also includes 28 subjects’ EEG data (14 SZ and 14 HC subjects). This dataset was obtained from 19 channels (Fp1, Fp2, F3, F7, F4, F8, C3, C4, T3, T4, T5, P3, Fz, Cz, Pz, P4, T6, O1, O2) at a sampling rate of 250Hz while the subjects were in resting state.

Details data collection process and description of ASD, EP, PD and SZ datasets can be found in [54], [55], [25] and [24], respectively. All of those datasets are available online and informed consent of the subjects was taken during the data collection time for publishing the data. Moreover, participants’ confidentiality is protected by not posting any personal identification information about the respondents, which is why, no ethical approval was required for our study.

### Classification performance measure

**Fig 3:**
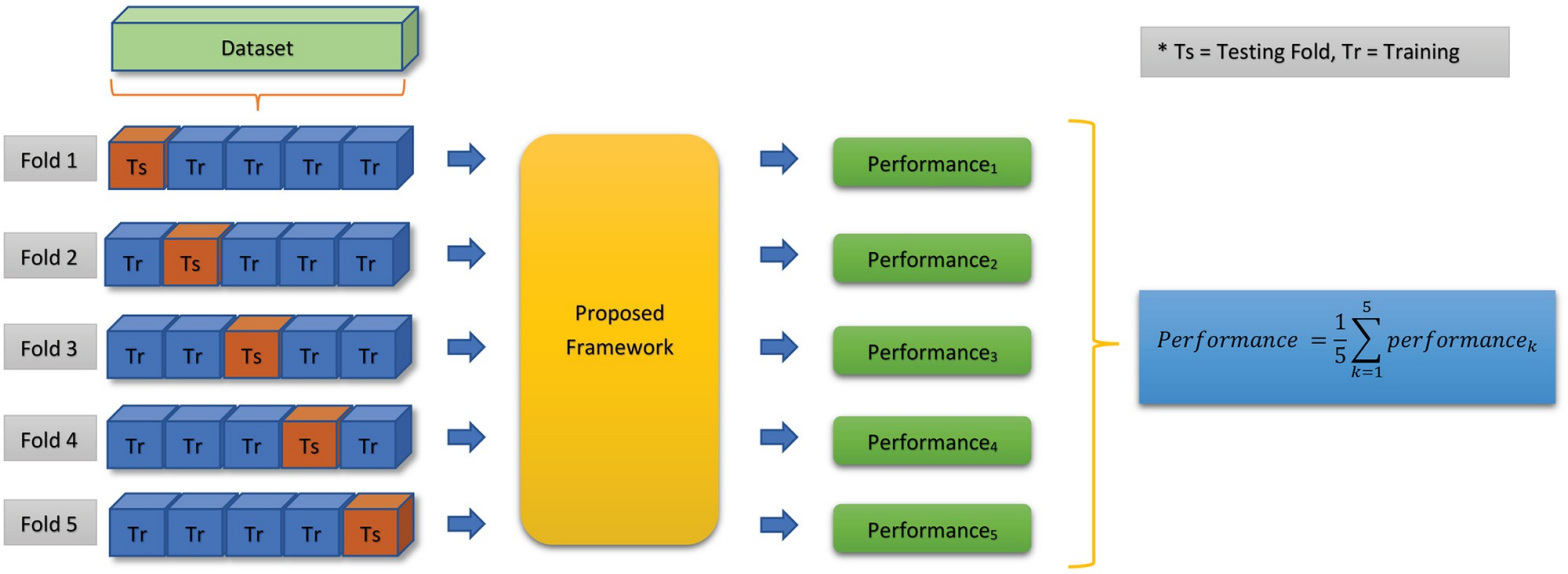
Five-fold cross validation technique used in this study.

To reduce the bias of the model’s classification performance and predict the overall accuracy of the model on the full dataset, a cross-validation scheme is recommended [6, 56–58]. In this study, we have used five-fold cross-validation technique to validate the performance of the proposed models. In this process, the dataset is arbitrarily divided into five equal or nearly equal parts and among those parts, four parts are used for training the classifier and the rest part is used for testing the trained system. This process is repeated five times so that each image of the dataset belongs to the test set exactly once. This testing process is depicted in Fig 3.

Finally, the generated results from the five-folds are used to evaluate the performance of the system using six parameters namely, sensitivity (Sen), specificity (Spec), precision (Prec), F1 score (F1), accuracy (Acc) and receiver operating characteristic (ROC) curve. These criteria allow to predict the behavior of the classifiers on the test data [23, 59, 60]. Four parameters namely True Positive (TP), True Negative (TN), False Positive (FP), and False Negative (FN) are used to calculate those six parameters using Eqs (11)–(15).

$$\begin{array}{c} Sensitivity\left(Sen\right)=\frac{TP}{TP+FN}*100 \end{array}$$ (11)

$$\begin{array}{c} Specificity\left(Spec\right)=\frac{TN}{TN+FP}*100 \end{array}$$ (12)

$$\begin{array}{c} Precision\left(Prec\right)=\frac{TP}{TP+FP}*100 \end{array}$$ (13)

$$\begin{array}{c} F1score\left(F1\right)=\frac{2\;TP}{2\;TP+FP+FN} \end{array}$$ (14)

$$\begin{array}{c} Accuracy\left(Acc\right)=\frac{\sum_{i=1}^{n}TP_{i}}{TP+FP+TN+FN}*100 \end{array}$$ (15)

**Fig 4:**
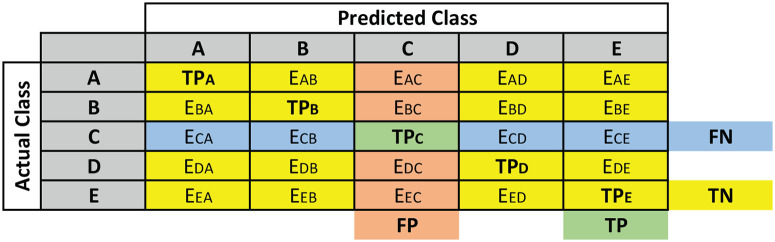
Confusion matrix of five class classification.

TP, TN, FP and FN can be defined for multi-class classification using the confusion matrix given in Fig 4. The figure shows the TP, TN, FP and FN values for class C, where green colored cell gives the TP value, blue cells sums up for FN, yellow colored cell sums up for TN and orange colored cells sums up for FP values. TP, TN, FP and FN values for other classes can be calculated in similar way.

The ROC graph is a handy tool for visualizing the classifier’s reliability, that is made by plotting sensitivity (true positive rate) on the Y-axis and 1-specificity (false positive rates) on the X-axis. These parameter can be used to predict how classifiers will act when dealing with test data [6, 23, 59, 61–63].

## Experimental results and discussion

In this study, we have developed a brain signal data mining framework using spectrogram images of the signal data and ML based approaches. The proposed framework was tested on four (ASD, EP, PD and SZ) neurological diseases related EEG datasets and performed a five class (ASD vs EP vs PD vs SZ vs HC) classification task. This section describes and visualizes the obtained results in detail with experimental setups.

### Experimental setup

From the EEG recording information, we found that the datasets have different sampling rates and various number of recording channels. Therefore, to make the datasets comparable we have to format the datasets into a common standard. To do so, we have selected minimum number of available channels among the datasets as the base dataset and converted all other datasets to that format. We kept the ASD dataset as base, as it has lowest number of recording channel and converted other three datasets (PD, EP, SZ) into that format by keeping data from standard 16 channels (Fp1, Fp2, F3, F4, F7, F8, C3, C4, P3, P4, T3, T4, T5, T6, O1 and O2) and discarding other channel data and finally, resampled them into a sampling rate of 256Hz.

After formatting the datasets, all the EEG data signals are pre-processed to remove noises and artifacts, and then segmented into 3 second time frame. After that, the signal segments are used to generate spectrogram image using STFT based spectrogram plotting technique. This produced a total of 19417 images from four datasets where ASD, EP, PD and SZ datasets contributed 5437 (3825 ASD, 1612 HC), 2483 (1248 EP, 1235 HC), 1745 (864 PD, 881 HC) and 9752 (5312 SZ, 4440 HC) images respectively. We merged all the HC images and formed a class of HC subjects with a total of 8168 images, producing a five class categorization problem (ASD vs EP vs PD vs SZ vs HC). These images are then used for feature extraction and ML based classification process.

### Results

In this proposed brain signal data mining framework, we have used two different histogram based techniques to extract textural features from the spectrogram images named cCENTRIST and tCENTRIST. Then PCA is used to reduce the dimension of the extracted features and finally, four ML based classification techniques SVM (LibSVM), RF, LDA and *k*NN (with *k* = 1 to 10) are used to perform classification of the reduced features for both extractors separately. Five-fold cross validation technique is used to validate the performance of the classifiers. Table 1 depicts the five round average results of the Eqs (11)–(15) for four classifiers. Here for *k*NN, we have only mentioned the results of *k* = 9 as it produced the best result among the ten different *k* settings we have tested.

**Table 1:** Average Sensitivity, Specificity, Precision, F1 Score and Accuracy over five rounds for two different feature extractors with four different classifiers.

**cCENTRIST based feature extraction**																
	**SVM**				***k*** **NN**				**RF**				**LDA**			
**Disease**	**Sen%**	**Spec%**	**Prec%**	**F1**	**Sen%**	**Spec%**	**Prec%**	**F1**	**Sen%**	**Spec%**	**Prec%**	**F1**	**Sen%**	**Spec%**	**Prec%**	**F1**
**ASD**	90.72	96.80	87.45	0.89	87.59	97.59	90.03	0.89	75.77	97.40	87.69	0.81	79.67	96.92	86.40	0.83
**EP**	83.59	98.51	79.46	0.81	77.14	98.06	73.29	0.75	31.87	99.97	98.56	0.48	80.70	97.19	66.36	0.73
**Normal**	84.77	87.83	83.49	0.84	88.89	86.56	82.77	0.86	92.29	70.17	69.20	0.79	78.69	86.33	80.70	0.80
**PD**	83.99	99.61	91.01	0.87	69.11	99.95	98.37	0.81	24.26	100.00	100.00	0.39	83.47	98.25	69.06	0.76
**SZ**	84.91	96.22	89.43	0.87	86.27	97.06	91.72	0.89	76.35	96.09	88.02	0.82	81.95	92.84	81.19	0.82
**Avg**	**85.59**	**95.79**	**86.17**	**0.86**	**81.80**	**95.85**	**87.23**	**0.84**	**60.11**	**92.72**	**88.69**	**0.66**	**80.90**	**94.31**	**76.74**	**0.78**
**Acc**	**85.87(± 0.45)**				**86.28(± 0.42)**				**77.76(± 0.53)**				**80.1(± 0.74)**			
**tCENTRIST based feature extraction**																
**ASD**	92.26	97.44	89.88	0.91	91.96	96.64	87.16	0.89	71.88	97.77	88.82	0.79	75.96	93.93	75.39	0.76
**EP**	86.16	98.96	85.04	0.86	78.74	98.10	73.99	0.76	23.25	99.92	95.45	0.37	74.37	95.92	55.59	0.63
**Normal**	88.77	89.50	85.99	0.87	88.76	89.49	86.00	0.87	95.96	62.74	65.15	0.78	68.47	83.64	75.24	0.72
**PD**	87.95	99.73	93.85	0.91	88.94	99.96	99.10	0.94	39.77	99.97	98.35	0.57	80.91	98.36	69.82	0.75
**SZ**	87.06	97.45	92.78	0.90	85.86	98.02	94.25	0.90	67.36	99.57	98.35	0.80	74.26	89.26	72.26	0.73
**Avg**	**88.44**	**96.61**	**89.51**	**0.89**	**86.85**	**96.44**	**88.10**	**0.87**	**59.64**	**91.99**	**89.23**	**0.66**	**74.79**	**92.22**	**69.66**	**0.72**
**Acc**	**88.78(± 0.36)**				**87.96(± 0.36)**				**76.21(± 0.45)**				**72.46(± 0.39)**			

**Fig 5:**
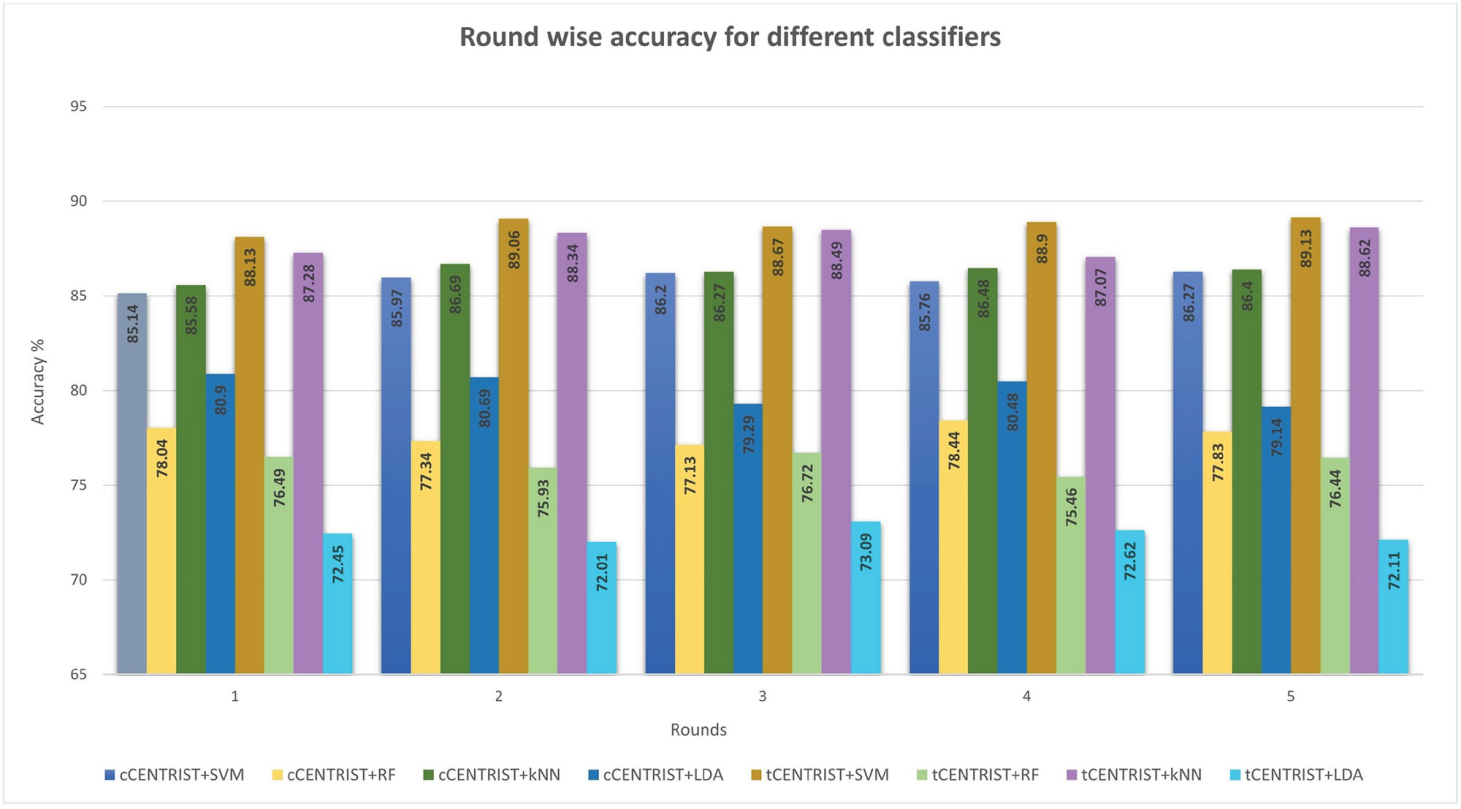
Round wise accuracy comparison for different classifiers.

From Table 1, we can see that, for cCENTRIST based feature extraction approach, *k*NN produces the overall best accuracy of 86.28% and RF gives the lowest overall accuracy among the four classifiers. SVM classifier produces nearly the same accuracy as the *k*NN while LDA produces moderate accuracy among the four. For a single round, highest accuracy is achieved 86.69% in round 2 for *k*NN and lowest accuracy is 77.13% for RF in round 3. For tCENTRIST based feature extraction process, SVM produces the highest overall accuracy of 88.78% and LDA generates the lowest overall accuracy of 72.46%. For *k*NN and RF, those values are 87.96% and 76.21%, respectively. For a single round, highest and lowest accuracy values are 89.13% and 72.01% using SVM and LDA, respectively. Round wise accuracy comparison for the different classifiers with two different feature extraction techniques are plotted in Fig 5.

**Fig 6:**
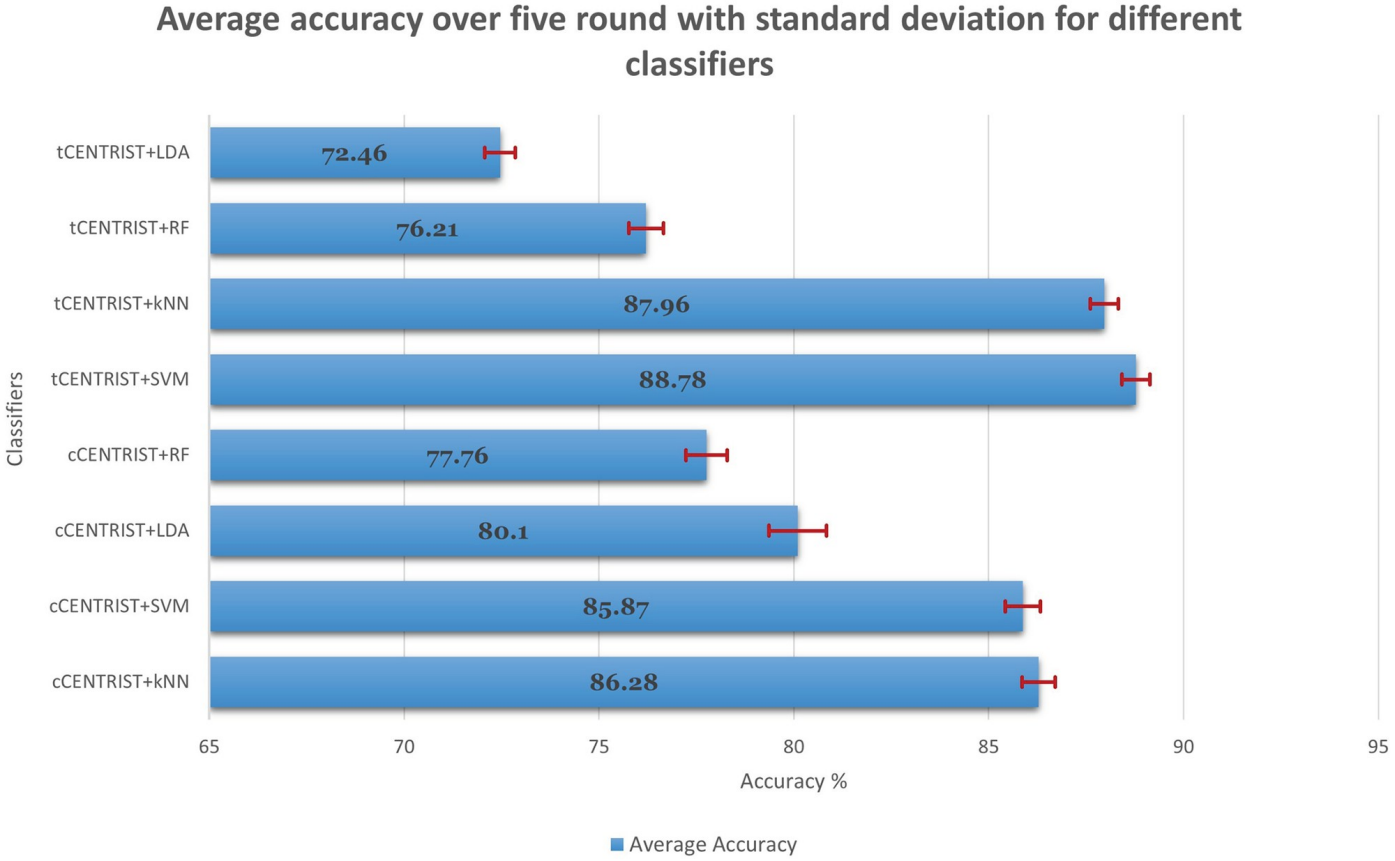
Average accuracy with standard deviation over 5-fold for different classifiers.

We have also compared the classifiers average accuracy over five-fold with standard deviation (SD) and plotted in Fig 6. Among the eight different classification (two different feature extraction techniques with four different classifiers) experimental results, SVM with tCENTRIST based feature extraction technique produces the highest average accuracy of 88.78% with a SD value of 0.36. On the other hand, LDA with tCENTRIST has the lowest average accuracy of 72.46% with SD value 0.39.

**Fig 7:**
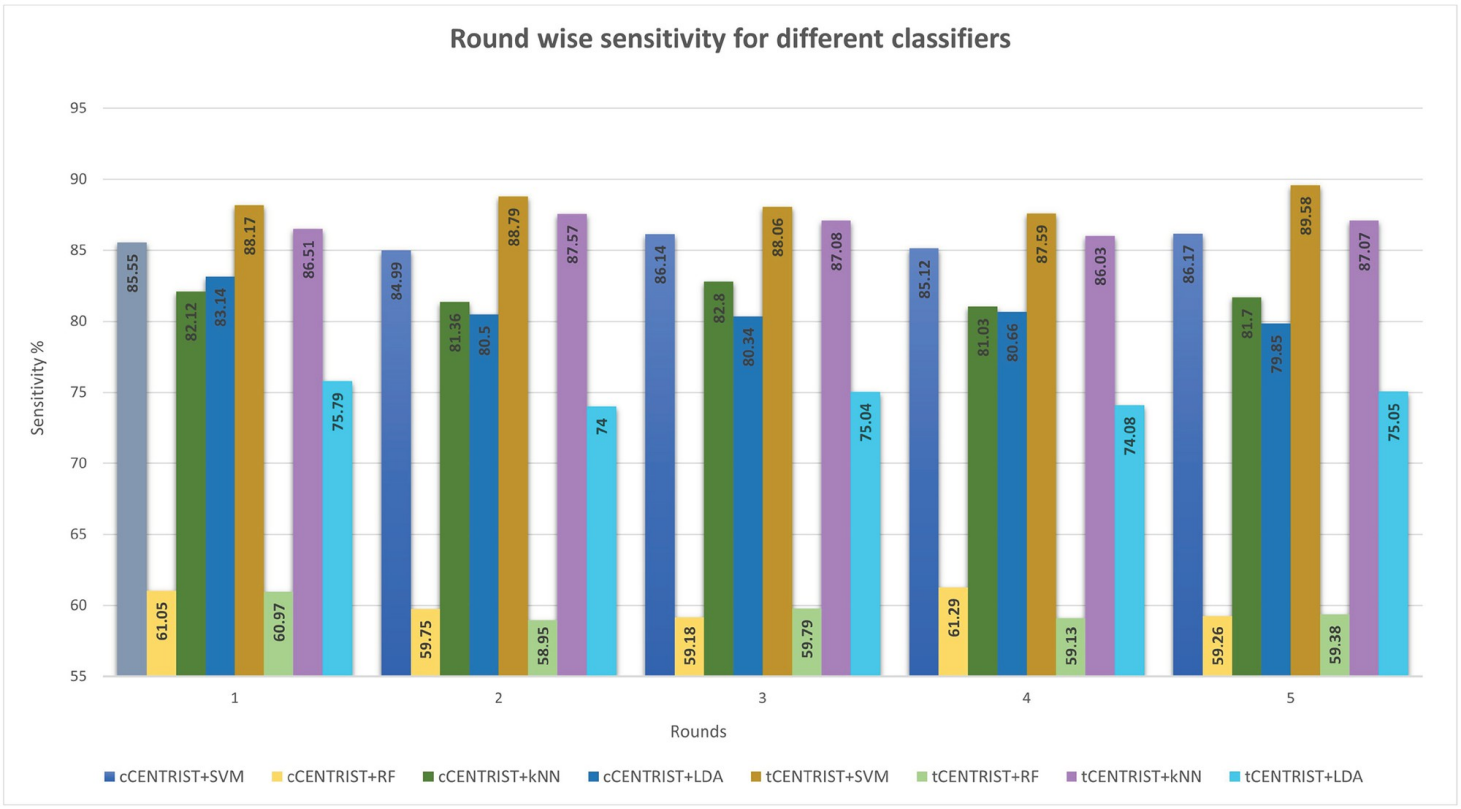
Round wise sensitivity comparison for different classifiers.

**Fig 8:**
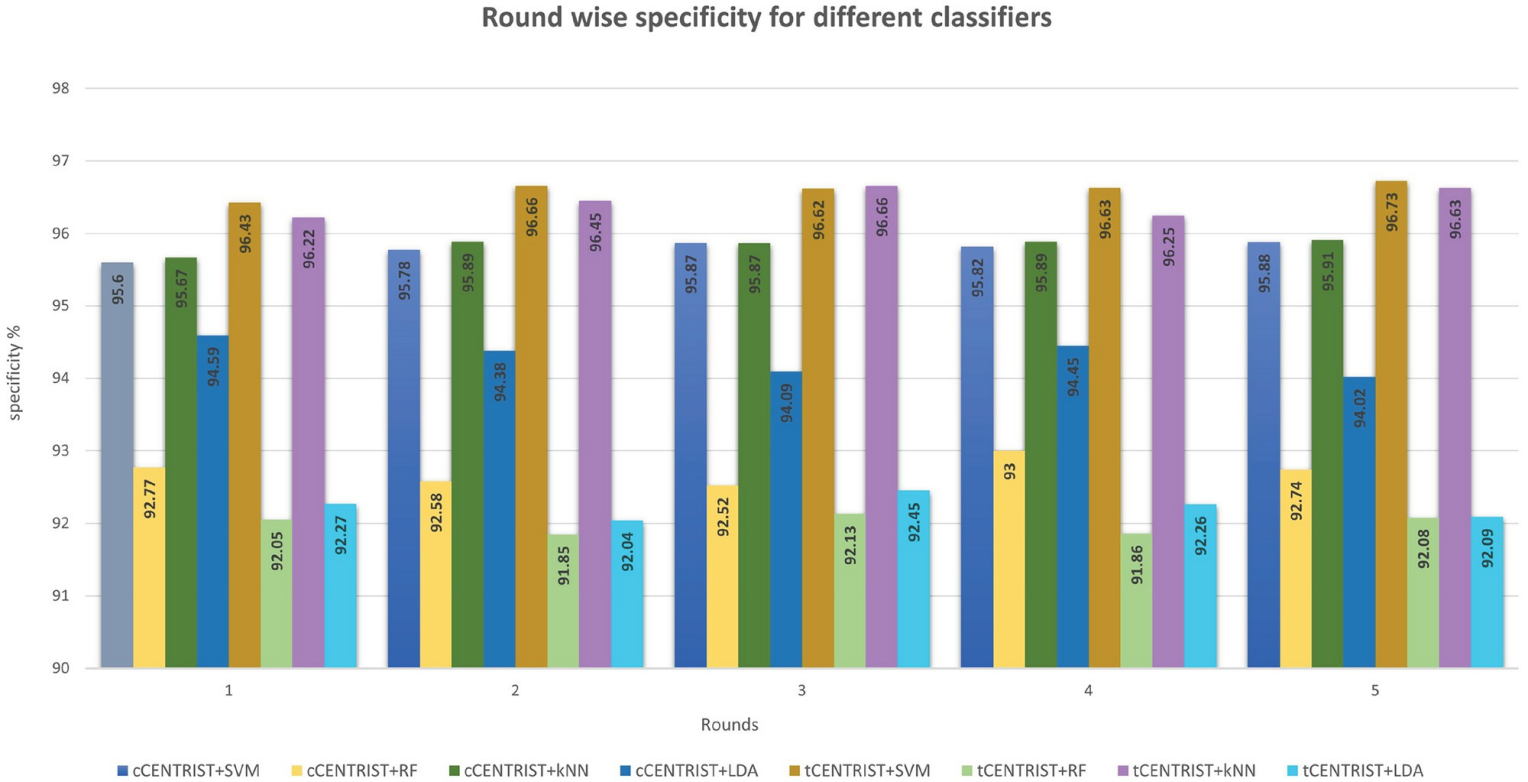
Round wise specificity comparison for different classifiers.

**Fig 9:**
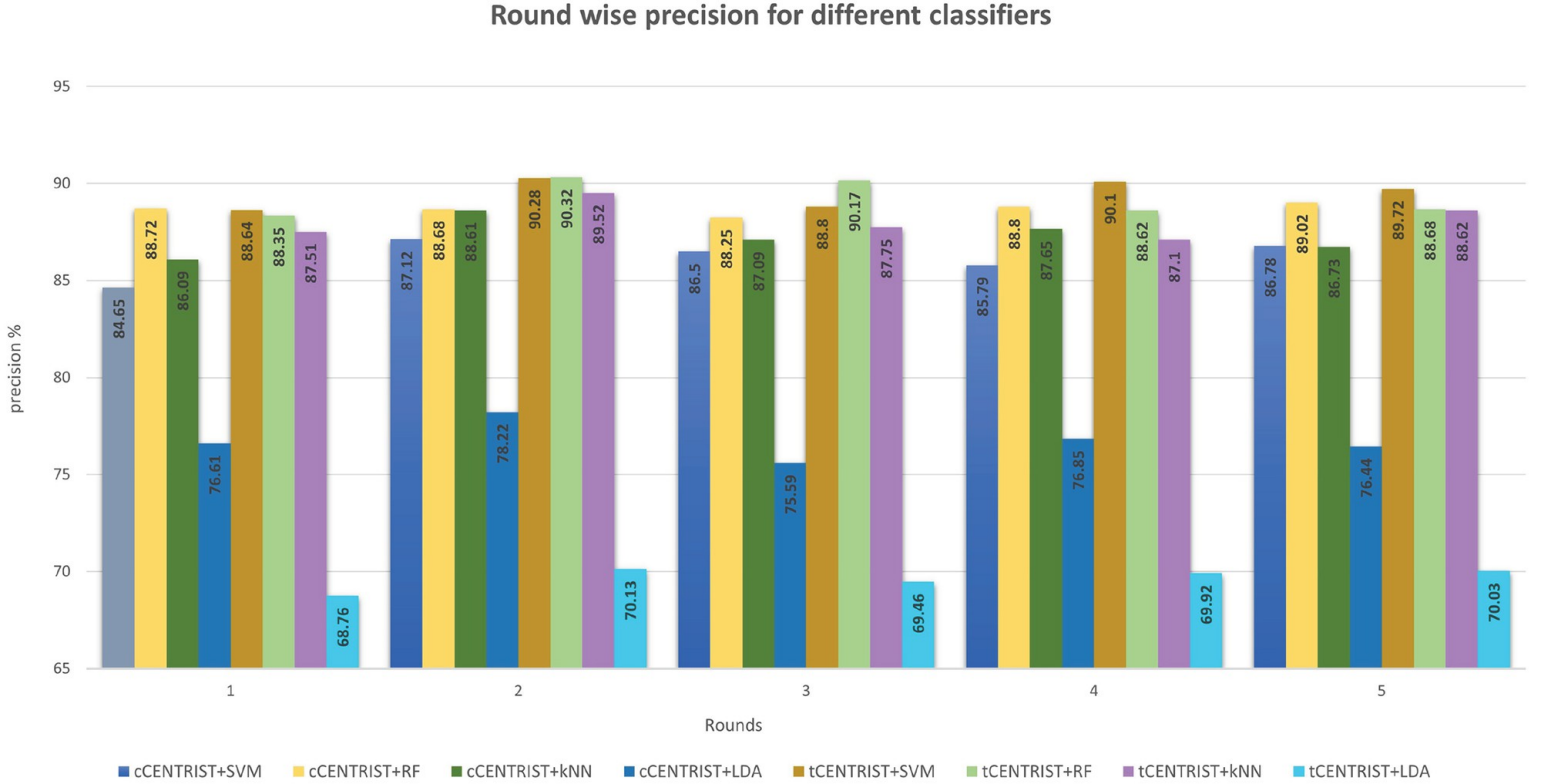
Round wise precision comparison for different classifiers.

**Fig 10:**
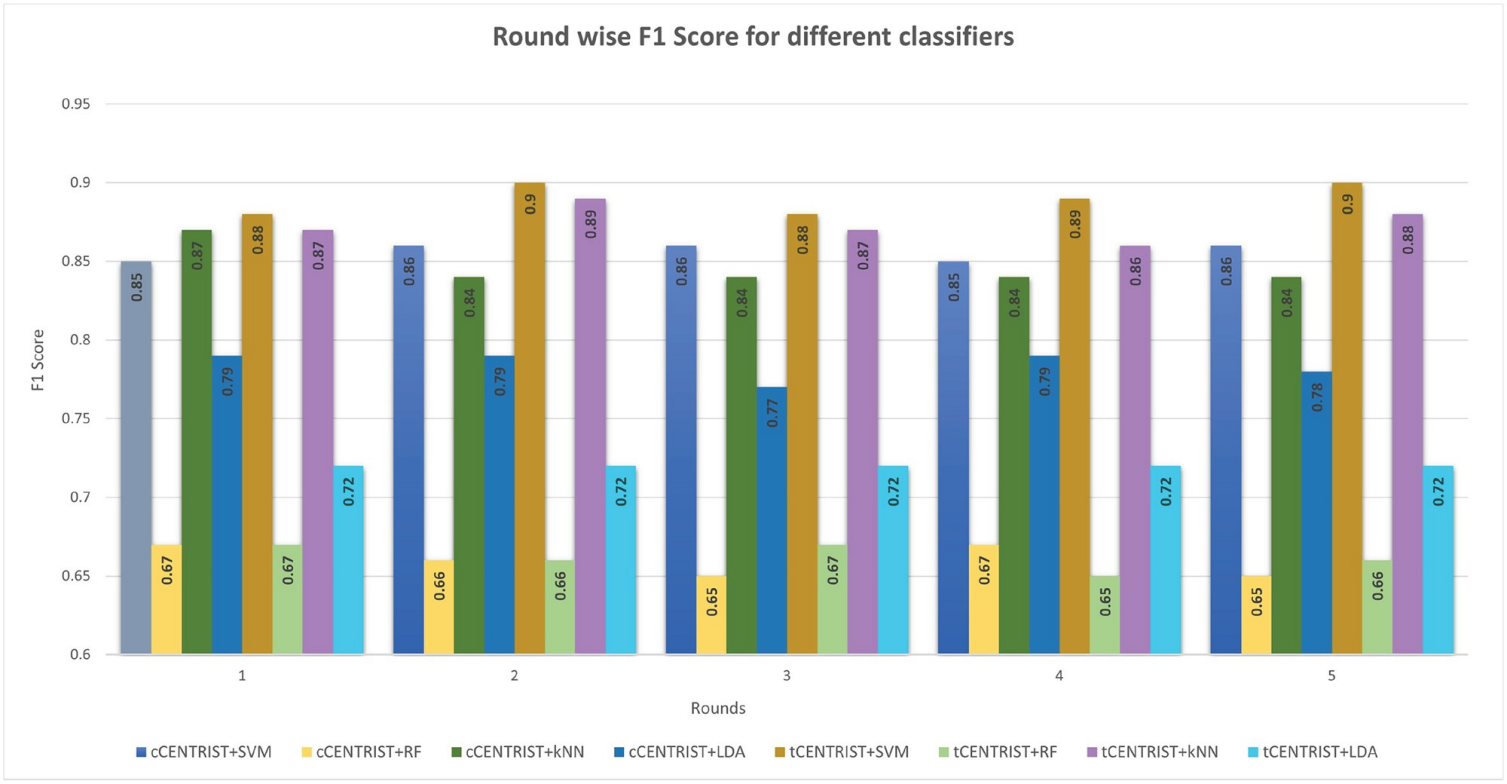
Round wise F1 score comparison for different classifiers.

To further assess the performance of the proposed classifiers, we have calculated and plotted the sensitivity, specificity, precision and F1 score for all the classifiers using Eqs 11–15 and have made some comparative visualization as shown in Figs 7–10.

From Fig 7, we can see that, tCENTRIST+SVM produces the highest single round sensitivity value of 89.58% and an overall 5-fold average value is 88.44% (SD 0.69). tCENTRIST+RF has the lowest single round sensitivity of 58.95% with 5-fold average value of 59.64% (SD 0.72). For cCENTRIST feature extractor, in case of 5-fold average value, SVM gives the highest sensitivity of 85.59% (SD 0.49) and RF gives the lowest value of 60.11% (SD 0.89). This result indicates that the tCENTRIST+SVM classifier is highly sensitive in detecting diseases than other classifiers, which is desired.

Fig 8 plots round wise specificity of the used ML based classifiers where we can see that both SVM and *k*NN have the similar specificity value over the rounds for both of the feature extractors. tCENTRIST+SVM produces both single round and five round average highest specificity values, which are 96.73% and 96.61% (±0.1), respectively. On the other hand, tCENTRIST+RF has the single and five round average lowest specificity of 91.85% and 91.99% (±0.12). Higher specificity value indicates the model’s ability to differentiate the healthy subjects from patients.

Precision is an important measure in information retrieval and classification framework evaluation which indicates the percentage of retrieved instances that are relevant. Fig 9 plots the round wise precision value for the different classifiers. From the plot, we can see that, although the RF classifier has very poor overall performance for both cCENTRIST and tCENTRIST, but its precision is high compared to other classifiers in most of the cases. This is because, even though its sensitivity is low but those images that it identified as patient’s image are mostly correct compared to other classifiers. Overall, five round average highest precision is produced by tCENTRIST+SVM which is 89.51% (±0.67), followed by tCENTRIST+RF with a value of 89.23% (±0.84). Lowest average precision value of 69.66% (±0.51) is produced by tCENTRIST+LDA.

F1 score is the harmonic mean of precision and recall, and Fig 10 depicts the round wise F1 score for the tested classifiers. It is also an important measure to assess the performance of the classifiers. From the plotting, we can see that SVM classifier outperforms other classifiers in all round values. Overall tCENTRIST+SVM has an average F1 score of 0.89 (±0.009) while for *k*NN, it is 0.84 (±0.005), and RF has the lowest average of 0.66 (±0.01).

**Fig 11:**
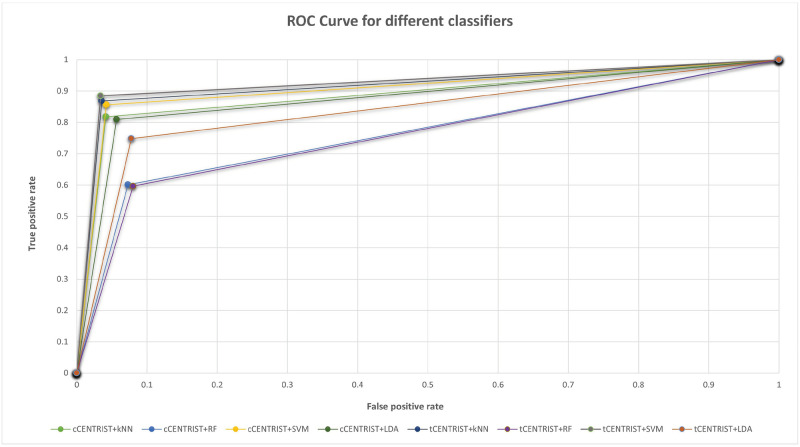
ROC graph comparison for different classifiers.

Finally, we plotted sensitivity on the y-axis and 1-specificity on the x-axis to construct the ROC curve for the classifiers. The ROC curve for the used classifiers are depicted in Fig 11. we can see that, the curve of tCENTRIST+SVM classifier is on top as it has the highest sensitivity among all the classifiers and tCENTRIST+RF has the lowest ROC curve as it has low sensitivity value.

### Discussion

In this study, we have used developed a framework for classifying multiple neurological disorder using spectrogram images of EEG data with textural feature extractor and ML based classifiers. EEG recordings from four different neurological disorders are used to validate the proposed system and have performed a five-class categorization task to validate it. Experimental results indicate that EEG biomarkers can be used to develop a single system for classifying multiple neurological disorders instead of using multiple binary classification system for individual diseases.

In addition, this concept of developing this five-class classification system was first introduced in our previous study [40], where we have used cCENTRIST with three ML based classifier and achieved an accuracy of 86.25%. In this study, we have extended that work with new textural classifier with additional one ML classifier and obtained an accuracy of 88.78%. Table 2 shows the comparison of this study with the existing studies that have done the same five-class classification task.

**Table 2:** Comparison of the proposed method with existing five-class classification task done on the same datasets.

Author	Feature extractor	Classifier	Accuray
Tawhid *et al*. [40]	cCENTRIST	*k*NN, SVM and RF	86.28%
This study	cCENTRIST and tCENTRIST	*k*NN, SVM, RF and LDA	**88.78%**

Finally, the performance of this spectrogram image based classification framework indicates that this system can be extended in future to incorporate more neurological disorders to increase the number of classes in categorization process. Moreover, there is still scope in improving the performance of the system which can be achieved by using deep learning based classification techniques.

## Conclusion

In this study, a single system is developed for multi-disease brain signal data classification using time-frequency spectrogram image and machine learning based data mining techniques. There is a lack of systems that can classify multiple diseases using a single framework. We have used EEG brain signal data for classification of multiple neurological disorders to fill the gap. At first, the EEG data are filtered for noise and artifacts removal and segmented into small chunks. Then T-F based spectrogram images are generated from those segments using STFT. Textural features from those images are extracted using two histogram based feature extractor named: cCENTRIST and tCENTRIST and PCA is used for reducing the dimension of the extracted features. Finally, *k*NN (with *k* = 1 to 10), SVM, LDA and RF classifiers are used for classifying those features into five classes (ASD vs EP vs PD vs SZ vs HC). Among the tested classifiers, tCENTRIST with SVM achieved the highest accuracy of 88.78% followed by *k*NN with 87.96%.

In future, deep learning-based models like convolutional neural networks (CNN) can be used to classify the generated T-F based spectrogram images for mining brain signal data and improve the classification performance. This is because deep learning models like CNN are more powerful in image classification task and widely used. Moreover, different pre-trained models can be used in future using transfer learning to perform classification as the size of the dataset is not large enough. Additionally, more diseases can be incorporated in the system to increase the number of classes in the classification task and scalability of the proposed framework.
